# Pediatric lung transplantation in the largest lung transplantation center of China: embarking on a long road

**DOI:** 10.1038/s41598-020-69340-0

**Published:** 2020-07-27

**Authors:** Bingqing Yue, Bo Wu, Ji Zhang, Hongyang Xu, Dong Wei, Chunxiao Hu, Jingyu Chen

**Affiliations:** Wuxi Lung Transplant Center, Department of Thoracic Surgery, Wuxi People’s Hospital Affiliated to Nanjing Medical University, Wuxi, 214023 China

**Keywords:** Respiratory tract diseases, Paediatric research

## Abstract

Lung transplantation (LT) has been an effective treatment for carefully selected children with end-stage lung diseases. The aim of this retrospective study is to introduce our experience at the largest LT center in Wuxi, China and to compare the outcomes of pediatric LT between children with idiopathic pulmonary arterial hypertension (IPAH) and other end-stage lung diseases. Ten pediatric patients undergoing LT from 2007 to 2019 were included. Sequential bilateral lung transplantation (BLT) with bilateral anterior thoracotomies was performed in all patients, seven of whom also underwent reduced size LT. Eight children survived until the end of our follow-up period on July 31st, 2019, with the longest survival of 11 years. Extracorporeal membrane oxygenation (ECMO) was intraoperatively used in all IPAH children and one non-IPAH child. Left heart function of IPAH children, though initially compromised, recovered after surgery. Statistically significant differences in operation time and post-operative mechanical ventilation between IPAH group and non-IPAH group were observed without discernible impact on post-LT survival. Pediatric LT appears to be a safe treatment for IPAH children to improve longevity and quality of life and ECMO may help reduce the risk of surgery and the postoperative complications.

## Introduction

The volume of adult lung transplants (LT) reports have increased notably in recent years with established evidence about indications, techniques, immunosuppressive drugs and criteria for adult lung donation. However, there is remarkable difference between the number of pediatric LTs and adult LTs worldwide. According to the 2018 International Society of Heart and Lung Transplantation (ISHLT), from June 2008 to 2017, 1,067 LTs were performed under the age of 18^1^, while 4,554 adult LTs occurred in the single year of 2016^2^. Specifically, the most common indication for pediatric LT in the western world is cystic fibrosis which is rarely reported in China^3, 4^. Other leading indications for pediatric LT consist of idiopathic pulmonary arterial hypertension (IPAH), bronchiolitis obliterans (BO) following bone marrow transplantation (BMT), surfactant dysfunction disorders and interstitial lung disease (ILD)^5^. The aim of this study was to share our experiences in pediatric LT conducted at our center, the largest specialized institution for LT in the whole country of China.

## Results

From 2007 to 2019, 10 pediatric LT recipients were performed with the median age of 14.9 years, ranging from 11 to 17. The average BMI of the recipients were 19.2 kg/m^2^ as shown in Table 1. Six children were diagnosed with IPAH, two with ILD and another two with BO following BMT. One child was hospitalized in the intensive care unit (ICU) for noninvasive ventilation before transplantation. However, no patients were on extracorporeal membrane oxygenation (ECMO) bridging before LT.

**Table 1:** General characteristics of child patients.

Case	Sex	Age (y)	Height (m)	Weight (kg)	BMI (kg/m^2^)	Blood group	Diagnosis	Preoperative sputum culture	Operation type	Reduced size LT	Intraoperative EMCO	Survival	Cumulative duration survival (days)
1	M	16	1.70	55	19.0	A	IPAH	N	Bilateral LT	N	Y	Y	4,248
2	M	14	1.60	50	19.5	AB	IPAH	N	Bilateral LT	N	Y	Y	2,481
3	F	17	1.60	55	21.5	A	IPAH	N	Bilateral LT	N	Y	Y	3,550
4	F	17	1.62	45	17.1	O	ILD	Y	Bilateral LT	Y	N	Y	2,044
5	F	13	1.61	44	17.0	B	IPAH	Y	Bilateral LT	Y	Y	Y	1,820
6	M	15	1.54	50	21.1	A	ILD	N	Bilateral LT	Y	N	Y	1,749
7	F	17	1.54	43	18.1	O	IPAH	N	Bilateral LT	Y	Y	N	26
8	M	13	1.54	45	19.0	A	BOS	N	Bilateral LT	Y	N	N	207
9	F	16	1.57	45	18.3	B	IPAH	Y	Bilateral LT	Y	Y	Y	1,072
10	M	11	1.47	47	21.8	O	BOS	Y	Bilateral LT	Y	Y	Y	34

*IPAH* idiopathic pulmonary arterial hypertension, *ILD* interstitial lung disease, *BOS* bronchiolitis obliterans syndrome, *ECMO* extracorporeal membrane oxygenation.

Sequential bilateral lung transplantation (BLT) with bilateral anterior thoracotomies was performed. Seven adolescents, accounting for 70%, underwent reduced size LT, including right middle lobe resection in 6 patients due to apparent oversized donor lungs. Intra-operative ECMO support was performed in 7 patients (6 with veno-arterial ECMO and 1 with veno-venous ECMO). The average operation time was 425 ± 74 min with the average blood loss volume of 1,700 ± 987 mL and blood transfusion volume of 1777 ± 790 mL (Table 2). Post-operative stay in ICU was 23 ± 30 days and patients were weaned from mechanical ventilation after an average of 13 ± 12 days.

**Table 2:** The surgery, outcomes and postoperative functional data of child patients.

	Mean ± SD	Range
**Intra-operative data**		
Blood loss volume(mL)	1,700 ± 987	1,000–3,500
Blood transfusion volume (mL)	1777 ± 790	1,150–3,800
Time (min)	425 ± 74	330–565
**Post-operative outcomes**		
ICU stay (days)	23 ± 30	2–103
Hospital stay (days)	51 ± 33	14–103
Mechanical ventilation time (days)	13 ± 12	1–33
**Lung function**		
FVC (L)	2.50 ± 0.83	1.45–3.55
%FVC (%)	62.34 ± 22.40	33.1–96.8
FEV1 (L)	2.10 ± 0.67	1.23–3.12
%FEV1 (%)	60.39 ± 19.98	32.8–85.8
FEV1/FVC	84.32 ± 4.56	77.31–91.54
MVV (L)	70.10 ± 19.24	53.69–108.66
%MVV (%)	55.47 ± 18.97	35.3–93.5
**Renal function**		
Cr (µmol/L)	64 ± 40	26–138
**Liver function**		
AST (U/L)	33 ± 26	13–91
ALT (U/L)	28 ± 13	12–44
TBIL (µmol/L)	27 ± 25	8–94

*ICU* intensive care unit, *FVC* forced vital capacity, *FEV1* forced expiratory volume in one second, *MVV* maximum ventilation volume, *Cr* creatinine, *AST* aspartate transaminase, *ALT* glutamic-pyruvic transaminase, *TBIL* total bilirubin.

**Figure 1:**
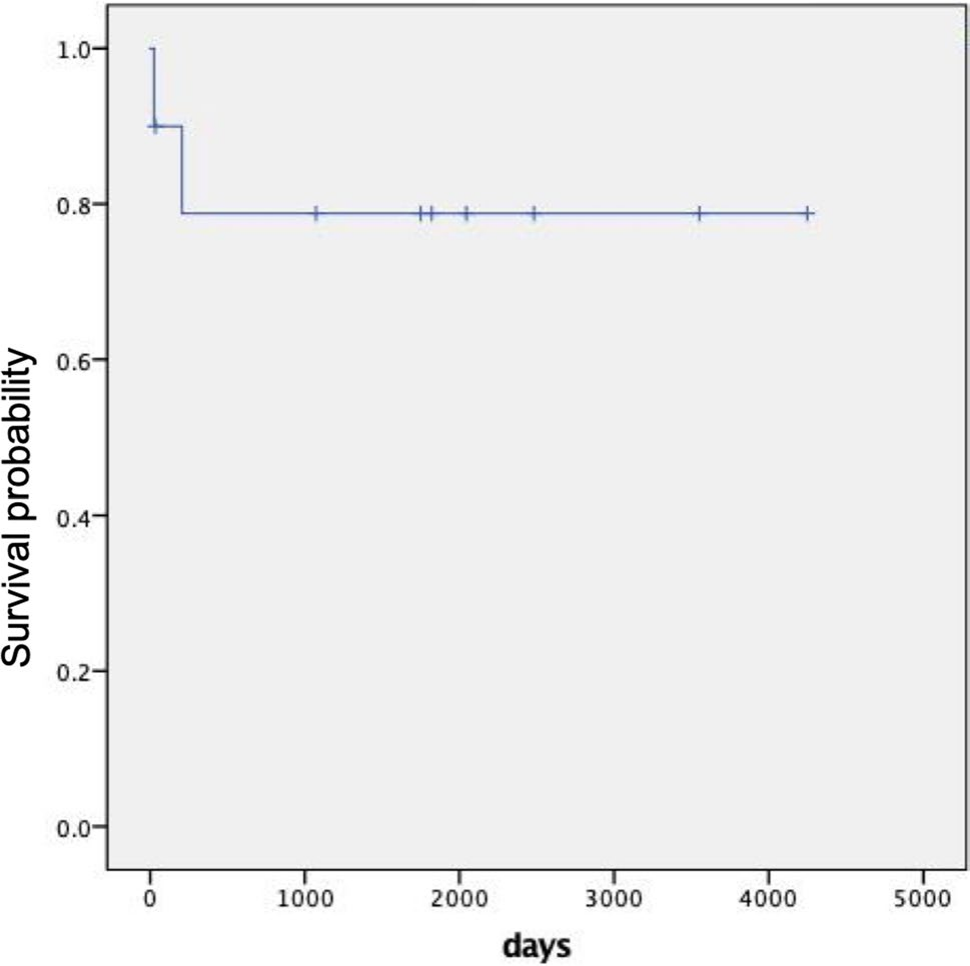
Kaplan–Meier plot for post-lung transplant survival for all cases.

Among the ten recipients, eight survived until the end of our follow-up on July 31st, 2019, with the mean survival time of 3,371.19 ± 552.43 days calculated by Kaplan-Meier method (Fig. 1). Death occurred in two cases, both within 1 year after LT. One patient developed acute left heart failure after surgery and required ECMO for maintenance treatment, which caused severe coagulapathy. The child later developed septic shock and his sputum was cultured as extensively drug-resistant *Acinetobacter baumannii*. He eventually died of airway bleeding 26 days after transplantation. Another child developed left anastomotic stenosis and right upper and middle bronchial stenosis with severe fungal and bacterial infection. We tried balloon dilation three times but failed. Despite the use of a ventilator, his oxygen saturation was still difficult to maintain. He developed severe hypoxemic respiratory failure and ischemic encephalopathy and finally died on the 207th day. In the first post-operative month, no significant abnormalities in liver and kidney function were observed and the lung function was acceptable. All indices were shown in Table 2.

Operative complications are summarized in Table 3. All children developed pulmonary infection during hospital stay. The most common pathogens were *Acinetobacter baumannii*, followed by *Pseudomonas aeruginosa* and *Klebsiella pneumoniae*. In the early postoperative stage, six patients suffered from acute left heart failure that mainly occurred in IPAH group (*n* = 5); all improved with conservative treatment. Two with postoperative ECMO underwent second operation due to pleural cavity hemorrhage on the first day after transplantation. Two developed Grade 3 primary graft dysfunction (PGD) and were treated with high-dose steroids and diuretics. One suffered from acute rejection according to computed tomography (CT) manifestations and positive panel reactive antibodies (PRA). The CT showed that her both lungs were scattered with light-density infiltrating shadows and her clinical symptom was ameliorated after high-dose steroids. During the follow-up period, one child with BO after BMT had intestinal graft-versus-host disease (GVHD) for the second time after LT. He experienced a significant decrease in platelets with diarrhea 4–6 times a day, accompanied with green mucous stool. The administration of methylprednisolone, mycophenolate mofetil, as well as gamma-globulin, relieved the child's symptoms. Two patients developed anastomotic stenoses after the surgery.

**Table 3:** Post-LT complications in child patients.

Complication	Numbers of patients
Infection	10
Acute left heart failure	6
Postoperative bleeding	2
Diabetes	2
PGD	2
Anastomotic stenosis	2
Renal dysfunction	2
Acute rejection	1
GVHD	1

*LT* lung transplantation, *PGD* primary graft dysfunction, *GVHD* graft-versus-host disease.

**Figure 2:**
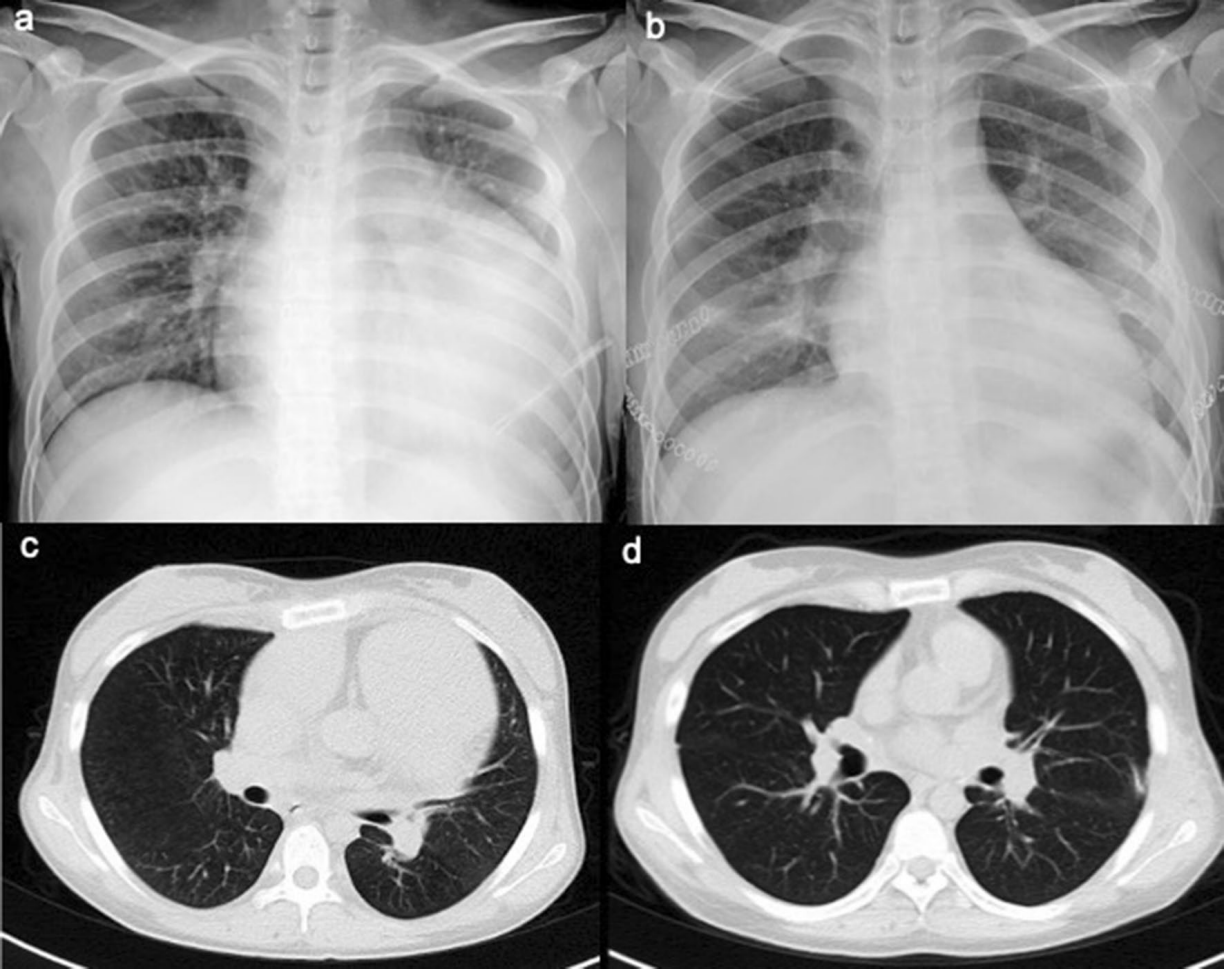
Radiogical examination. a, b: A chest radiograph of a child patient with IPAH was taken at 19 days before surgery (**a**). The chest radiographs of the IPAH child were performed one month after surgery (**b**). The CT examination of the IPAH child was implemented 5 months before surgery (**c**). The CT examination of the IPAH child was carried out 2 months after surgery (**d**). Comparisons of preoperative and postoperative images revealed significant improvement in IPAH after surgery.

Postoperative cardiovascular morphological changes in IPAH patients are shown in Table 4. To illustrate, left ventricular diameter at end diastolic phase and left atrial diameter within one month after the surgery were significantly larger than preoperative ones with a statistically significant difference. Stroke volume also increased significantly, which means left ventricular function was restored to some extent. Comparison between preoperative and postoperative chest radiographs and CT images in an IPAH child demonstrated significant improvement in pulmonary hypertension after transplantation (Fig. 2).

**Table 4:** Preoperative and postoperative cardiac ultrasound examination of cardiovascular morphology in IPAH children.

	Pre-LT	Post-LT	*P*
Aortic root diameter (mm)	24.75 ± 1.50	27.25 ± 1.50	0.194
Left atrial diameter (mm)	24.50 ± 2.52	32.25 ± 2.87	0.014^a^
Left ventricular diameter at end diastolic phase (mm)	31.75 ± 6.50	44.00 ± 2.94	0.018^a^
Interventricular septum thickness (mm)	8.00 ± 1.41	9.00 ± 2.16	0.572
Thickness of the left ventricle at posterior wall (mm)	8.00 ± 1.41	8.75 ± 2.36	0.697
Stroke volume (ml)	24.57 ± 5.17	50.90 ± 5.52	0.024^a^

*LT* lung transplantation.

^a^This symbol indicates *P *value < 0.05 based on the t-test.

The average operative time in IPAH cases, of 466 ± 55 min, was significantly longer than that in non-IPAH group, of 364 ± 53 min (*P* = 0.019). Days of post-operative mechanical ventilation period differed in the two groups with 20 ± 9 days in IPAH group and 2 ± 1 days in non-IPAH group (*P* = 0.006). There were no differences in blood loss volume, blood transfusion volume, ICU stay and total hospital stay between the two groups. ECMO was intraoperatively used in all IPAH patients and one BO patient with statistically significant (*P* = 0.033). There was no significant difference in postoperative complications and survival between the two groups (Table 5).

**Table 5:** Comparisons of intraoperative and postoperative characteristics between IPAH children and non-IPAH children.

	IPAH	Non-IPAH	*P*
**Intra-operative data**			
Bleeding volume(mL)	1867 ± 1,088	1,450 ± 900	0.545
Blood transfusion volume (ml)	1888 ± 981	1556 ± 426	0.548
Time (min)	466 ± 55	364 ± 53	0.019^a^
Intraoperative EMCO	6	1	0.033^b^
**Post-operative outcomes**			
ICU stay (days)	34 ± 35	6 ± 5	0.145
Hospital stay (days)	55 ± 34	44 ± 36	0.656
Mechanical ventilation time (days)	20 ± 9	2 ± 1	0.006^a^
Postoperative ECMO	5	1	0.19
Postoperative tracheotomy	4	0	0.076
Survival	5	3	1
Acute left heart failure	5	1	0.19
PGD	2	0	0.467
Postoperative bleeding	2	0	0.467
Anastomotic stenosis	1	1	1

*IPAH* idiopathic pulmonary arterial hypertension, *ECMO* extracorporeal membrane oxygenation, *PGD* primary graft dysfunction.

^a^This symbol indicates *P *value < 0.05 based on the t-test.

^b^This symbol indicates *P *value < 0.05 based on Fisher’s exact test.

## Discussion

Pediatric LT is an accepted option for a carefully selected subset of children with end-stage lung diseases for extending their survival and improving the quality of life^6^. However, only a few centers, mainly in North America, Europe and Australia perform pediatric LT. In recent years, successful pediatric lung transplants have also been reported in Asia^7, 8^. In the present study, we described a 10-case experience of pediatric LT in our center in the past decade. Despite the low clinical volume of pediatric LT in this series, they appear to have a longer survival after transplantation than adult patients^8^. In our study, children who survived more than 1 year are all alive now, with a 1-year survival rate of 80%.

In the reported clinical experience of pediatric LT in western countries, cystic fibrosis is the predominant indication in children aged over 5, while pulmonary hypertension (accounting for 50.4%) in those aged 1 to 5, and pulmonary hypertension (accounting for 38.1%) and pulmonary surfactant dysfunction disorders (accounting for 20.6%) in infants^1^. However, for adult LT recipients, ILD and chronic obstructive pulmonary disease are most common, and the proportion of adult patients with cystic fibrosis has declined in recent years^2^. In our center, more than half of the children were diagnosed with IPAH, two with BO and two with ILD respectively. BLT is the most common operative approach in pediatric LT in the world. During the past decade, the number of single lung transplantation in children worldwide accounts for only 4% of total pediatric LT cases^1^. Similarly, in our study, all cases received BLT. Evidence suggests that patients receiving BLT appear to have longer survival and better functional outcomes after transplantation^9, 10^.

**Figure 3:**
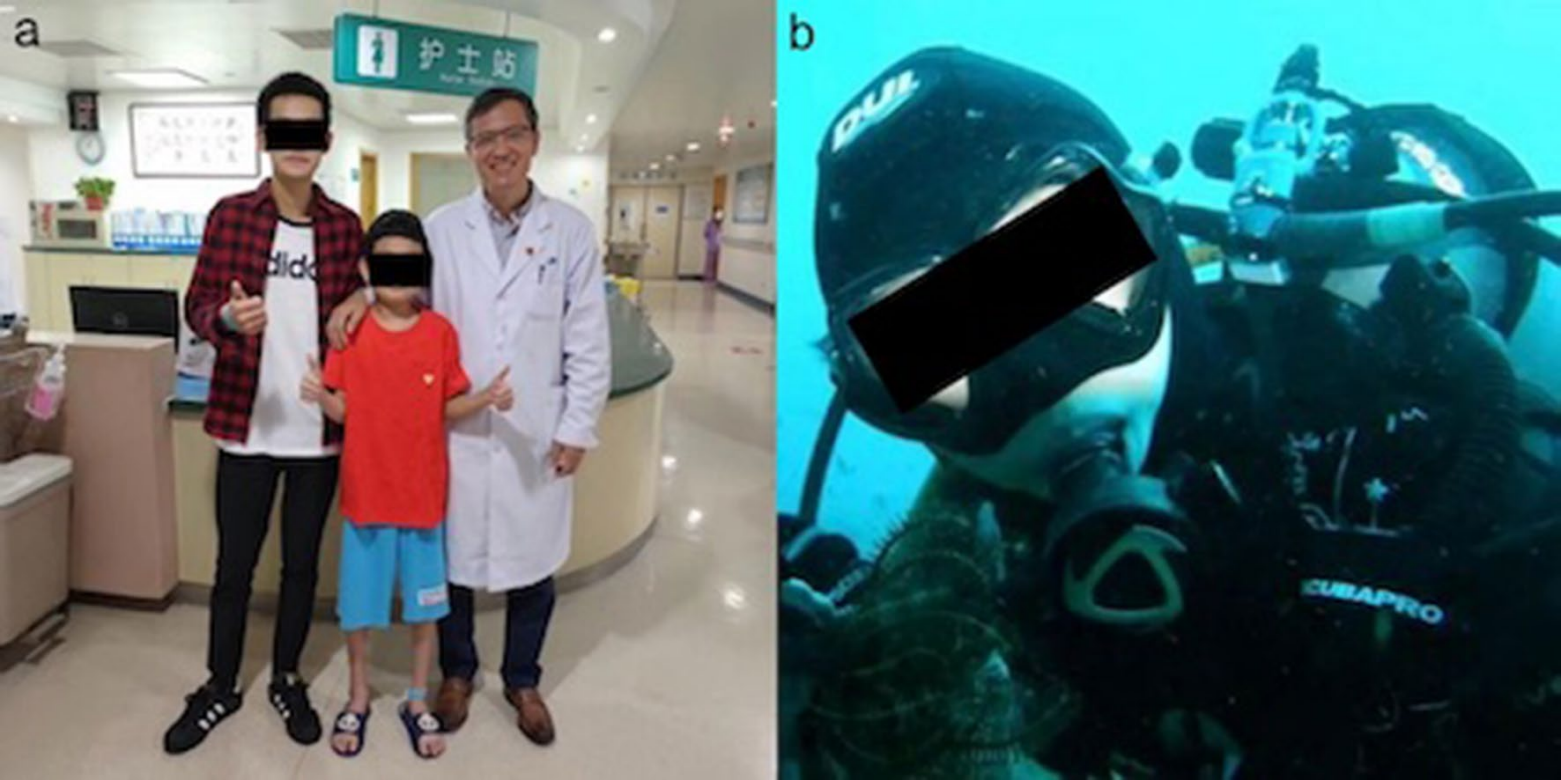
Life images of child patients. The boy in the middle of the photo suffered from BO and underwent BLT. He took this photo before discharging from our hospital (**a**). The girl who suffered from IPAH and underwent BLT in our center achieved a survival for 10 years and she took this diving photo about 1 year after transplantation (**b**).

At present, the most commonly used surgical incision of pediatric LT in western countries is transverse sternotomy via a clamshell incision or median sternotomy incision; while bilateral anterior incision is adopted in all cases of our center. In comparison with the clamshell incision, bilateral anterolateral incision is smaller and less traumatic. It reduces complications related to sternal healing, which means faster recovery and less pain. This year, we performed a pediatric LT for an 11-year-old child who was the youngest recipient in our center at that time. He recovered and was discharged from hospital only 14 days after the operation (Fig. 3a). Marczin et al. also conclude that patients receiving minimally invasive lung transplantation (MILT) seem to have better lung function in the early postoperative duration than those undergoing traditional transverse sternotomy, although the operation and management may be more complex^11^.

Currently the number of pediatric donors is very limited in our country, and it is difficult for the patients to find a commensurate donor lung for smaller thoracic cavities. As reported in the literature, adult donor lungs may be chosen for pediatric LT, especially older children and adolescents. Reduced size LT has been widely used in Europe in these years, especially for adolescents with cystic fibrosis^12^. Meuller et al. report that the prognosis is similar in those receiving either reduced size LT or full size LT, without significant differences in complications, survival and lung function between the two groups^13^. Therefore, reduced size LT may be a safe option for children with progressive disease without suitable donors. In our center, seven children underwent reduced size LT because of size mismatching. One month after surgery, all presented favorable lung.

Infection is a common complication especially in pediatric LT cases^14^, and is one of the important risk factors leading to early death after transplantation with the infection rate of 69–90%^15^. Onyearugbulem et al. retrospectively analyzed 98 lung transplant recipients in their center, 22 of whom developed infection after surgery^16^. Given the use of immunosuppressive agents, the treatment for post-LT infection can be challenging. Inevitably, pathogens were found in the sputum culture of all patients in our study. They had obvious symptoms of cough with sputum and their CT and bronchoscopy showed signs of pulmonary infection. Acinetobacter is the most common pathogenic bacteria in ICU in China with high drug resistance. Long ICU stay and mechanical ventilation time, preoperative infection, immunosuppressive drugs and repeated invasive bronchoscopy may increase the risk of infection after transplantation. One patient developed septic shock with extensively drug-resistant *Acinetobacter baumannii* infection and multi-organ dysfunction, finally dying of airway bleeding. The others were successfully treated and discharged. Another serious complication is airway complication which also frequently occurs after pediatric LT. The incidence of airway compliance in pediatric lung transplant recipients was reported to be similar with that in adult lung transplant recipients^5^. In our study, two patients had anastomotic stenosis after LT. One child developed right bronchial anastomotic stenosis. After six times of balloon dilation, her symptoms improved with FEV1 increasing from 1.23 to 1.91. Another child developed left anastomotic stenosis and right upper and middle lobe bronchial stenosis with severe fungal and bacterial infection. Despite of three times of balloon dilation, he eventually died. Acute rejection is a common complication in the early postoperative stage. Acute rejection is a common complication in the early postoperative period. According to ISHLT, the incidence of acute rejection within 1 year after lung transplantation in children is about 29%, higher than that in infants (3%); moreover, the incidence is higher in children aged 11–17 (35%)^1^. In our study, acute rejection appeared to only one child in the first year after surgery.

In the late phase, the main serious complications are BOS and posttransplant lymphoproliferative disorders (PTLD). Long-term use of drugs after surgery may lead to renal dysfunction and diabetes. The incidence of renal dysfunction, diabetes and BOS was 2%, 18.8% and 9.3% respectively 1 year after lung transplantation, and 6.1%, 28.6% and 36.9% respectively 5 years after lung transplantation^1^. The incidence of malignancy after lung transplantation is 5.6% and 11.2% respectively 1 year and 5 years after surgery, and the majority is post-transplant lymphoproliferative disease^5^. None of our children patients had BOS and PTLD. The incidence of diabetes and renal dysfunction was 10% in the 1st year and 12.5% in the 5th year after surgery.

ECMO, though, is adopted in some adults’ peri-LT stage to reduce the risk of complications^17^, particularly in patients with pulmonary hypertension, only a few articles report its use in pediatric transplantation^18, 19^. Whether it can be used as a bridge to transplant or be used intra-operatively and post-operatively with acceptable complication rates in children is still unproven. Toprak et al. show that the 1-year survival rate of pediatric patients who receive ECMO as a bridge for LT was 67%, which was not significantly different from that of the preoperative use in both mechanical ventilation group and the control group^18^. Zuercher et al. have performed intra-operative ECMO in 7 patients and they all have survived in the 3-month follow-up after surgery^19^. In our cohort, as more than half of our patients presented with severe pulmonary hypertension, the perioperative risk became very high. In order to maintain circulatory stability and reduce the risk of surgery, ECMO support was adopted in all IPAH patients, which likely explains the longer operative time compared with the non-IPAH group. Moreover, the critical status and undesirable coagulation function before transplantation result in exuberant bleeding of patients during ECMO, this is the main reason why two children in our center required further surgery to stop bleeding.

IPAH is a rare and progressive disease with unknown etiology and unfavorable prognosis, and the average survival time without targeted pharmacotherapy is only 2.8 years^20^. LT is the only effective method to cure the disease and prolong survival in patients with severe IPAH. In our study, we noted that left heart function of the patients recovered within one month after the surgery. Huis in't Veld et al. reported that compared to patients suffering from heart failure with preserved ejection fraction, patients with IPAH had much smaller left atria and significantly lower LA/RA ratios^21^. Gan et al. also found that PAH patients had decreased stroke volume, left ventricular end-diastolic volume, and left ventricle’s peak filling rate compared with normal people, and the decreased stroke volume was promoted by the impaired left ventricular filling function due to ventricular interaction mediated by the interventricular septum^22^. The reason may be that pulmonary hypertension caused enlargement of the right heart, leading to ventricular septal displacement and diastolic dysfunction of left ventricle. After the surgery, cardiac output would increase and left atrium and ventricle had the opportunity to fill and dilate because of the great decrease in the RV’s size. The child in Fig. 3b suffered from IPAH. She lived for 10 years after surgery and just got married. She took this photo about a year after transplantation while diving.

Patients with IPAH may have a higher perioperative risk. Our previous study has showed that high frequencies of syncope, hyponatremia, low cardiac index (CI), reduced inner diameter of left ventricle, and upward RV/LV ratio may increase perioperative risk in IPAH patients^23^. Sabashnikov et al. have reported that the length of ICU stay and hospital stay in IPAH patients is much longer than that in the control group; and the cumulative survival rate in the IPAH group is significantly lower in the 6-year follow-up^24^. In the early postoperative period, due to the sudden decrease in pulmonary artery pressure and right ventricular afterload, left ventricular diastolic function cannot be improved immediately, which may cause hemodynamic dysfunction and acute left heart failure. Moreover, pulmonary hypertension is the most clearly defined predisposing factor leading to PGD^25^. In our cohort, IPAH patients had longer mechanical ventilation time because of hemodynamic instability after surgery. The incidence of postoperative complications in IPAH patients was indeed higher than that in the non-IPAH group, due to hemorrhage, left heart failure and PGD.

Limitations of the current study encompass the small sample size and the retrospective nature of the study limiting the generalizability of our findings. We hope that these findings will permit an increased in pediatric volumes in Asia permitting long-term follow-up studies and serial quality improvement.

In conclusion, pediatric LT is an effective treatment for selected children with end-stage lung diseases, extending survival and ameliorating quality of life and cardiopulmonary function. Overall, the long-term prognosis of LT in children is similar to that in adults, and no obvious deterioration to other organs in the early postoperative stage has been observed. For our patients with IPAH, their quality of life following pediatric LT has significantly improved; However, the perioperative risk still remains high. ECMO may help reduce the risk of surgery and postoperative complications. Although a dearth of size-matched donors for children remains a problem in our country, similar therapeutic effects can be achieved by reduced size LT. At present, pediatric LT related case reports in China are rare. We believe that it is time to proactively promote pediatric LT in China to save more children with end-stage lung and pulmonary vascular disease.

## Methods

### Data collection of demographics

A retrospective analysis of the medical records of patients < 18 years old undergoing lung transplantation at Wuxi Center from 2007–2019. Patients were followed up until July, 2019. Ten candidates received LT in this period of time. Information collected included demographic data, clinical and laboratory results. For their condition evaluation, pre-surgery regimen, 6-min walk test (6MWT), spirometry test results, echocardiography and electrocardiogram were also performed. Post-surgery data encompassed complications, lung function, echocardiography, survival and death. The ABO blood groups of the donors and recipients were identical before operation. Preoperative chest X-ray or chest CT examination did not find any pulmonary infection or other pulmonary diseases in the donors, and the oxygenation index was > 300 mmHg.

The Institutional Ethics Committees of Wuxi People’s Hospital approved the study, including our retrospective review, verbal consent procedure and data analysis. All patients in our study were anonymous. Informed consents including publish their information or images in an online open-access publication for scientific research were obtained from the patients or their next of kin. The research was conducted in accordance with the 2000 Declaration of Helsinki and the Declaration of Istanbul 2008. None of the transplant donors were from a vulnerable population, and all donors or next of kin provided written informed consents that were freely given.

### Surgical technique

Seven patients underwent ECMO implantation prior to LT. In V-V mode ECMO arteriovenous pipelines were placed in the femoral vein and the internal jugular vein, and in V-A mode they were in the femoral artery and the femoral vein. The flow rate was controlled at 2–3.2 L/min.

All patients underwent MILT via bilateral anterior incision. First, the operation was started at an incision in the right fifth intercostal space and opened the right chest. We successively separated the right superior pulmonary vein and the right lower pulmonary vein, and then cut them in the pericardium. The right pulmonary artery was clamped with non-invasive vascular forceps and then dissected. The right main bronchus was dissected from the surrounding tissues. Then right lung was successfully explanted. The right main bronchus was anastomosed end-to-end by a 4–0 polydioxanone (PDS) running suture. The anastomotic stoma was embedded with interrupted sutures. The right pulmonary artery was anastomosed end-to-end with a 5–0 Prolene, followed by venous anastomosis with a continuous 4–0 Prolene suture. The suture was left untied anterior to the midpoint of the venous anastomotic stoma and secured after de-airing. We ventilated the right lung and opened the pulmonary artery clamp slowly. Then the patient was turned over and the left LT was performed via the same approach.

### Postoperative management

All children were transferred to the ICU after LT. They were currently managed by protective mechanical ventilation and antibiotics in case of infection. Anti-rejection therapy utilized standard triple immunosuppression of mycophenolate mofetil, tacrolimus, and corticosteroids. Blood gas analysis, chest radiographs and bronchoscopy were implemented regularly.

### Statistical analysis

All data were analyzed with SPSS statistical program version 23.0 (SPSS Inc., Chicago, IL). Descriptive statistics were used to analyze patient characteristics. Normally distributed continuous data were described as mean ± SD and analyzed by *t*-test. Categorical variables were calculated by Fisher’s exact test. Medians and ranges were presented for skewed data.

## Data Availability

The datasets generated during and/or analyzed during the current study are available from the corresponding author on reasonable request.
